# Antipredator Response in Domestic Japanese Quail and Game-Farmed Quail

**DOI:** 10.3390/ani15152237

**Published:** 2025-07-30

**Authors:** Pedro González-Redondo, Natalia Diego-Fuentes, Carlos Romero

**Affiliations:** 1 Departamento de Agronomía, Universidad de Sevilla, Carretera de Utrera km 1, 41013 Seville, Spain; nataliadifu@gmail.com; 2 Facultad de Ciencias y Artes, Universidad Católica Santa Teresa de Jesús de Ávila (UCAV), Calle Canteros s/n, 05005 Ávila, Spain; carlos.romero@ucavila.es

**Keywords:** antipredator behaviour, *Coturnix* spp., gamebirds, game farming, hunting quality

## Abstract

Game-farmed quails that are currently raised in captivity and released in hunting preserves are not attractive for many hunters because of their low antipredator instinct, which is due to the fact that in most cases, these farm-reared quails are hybrids between European common (*Coturnix coturnix*) and Japanese (*Coturnix japonica*) quails, with the latter having been selectively bred for docility. This study aimed at assessing the antipredator response of game-farmed and Japanese quails by performing three tests: human approach test, simulated aerial predator approach test and tonic immobility test. Thirty game-farmed quails (average body weight: 133 g) and thirty Japanese quails (323 g) were subjected to the tests. For each genotype of quail, fifteen males and fifteen females were used. In the human approach test, the distance at which quails moved was greater for game-farmed quails than for Japanese ones (37.4 vs. 19.6 m, *p* < 0.001). In the simulated aerial predator approach test, female quails of the Japanese species crouched down at the longest distance with respect to the predator (9.83 m), whereas no significant difference existed for this trait among the other three groups (6.84 m, on average). The percentage of quails flying when the predator got closer was higher for the Japanese species than for the game-farmed quails (23.3 vs. 3.33%, *p* = 0.023). Fewer inductions were needed to cause tonic immobility in the game-farmed quails than in the Japanese ones (3.10 vs. 4.10, *p* = 0.009), but then, the duration of the tonic immobility response did not differ significantly between the two genotypes. No effect of sex was detected in the human approach and tonic immobility tests. In conclusion, as compared with Japanese quails, game-farmed quails showed more fearful behaviour when confronted with a human being.

## Simple Summary

Currently, game farms usually raise a so-called game-farmed quail, which generally results from hybrid quails (*Coturnix coturnix* × *Coturnix japonica*) that are then backcrossed with males of the European common species (*Coturnix coturnix*). These farm-reared quails are being released in hunting preserves so as to counterbalance the decline of the wild stock of European common quails. However, the birds released are not really appreciated by hunters because of their calmness and poor ability to fly, which these birds owe to the fact of descending from domestic Japanese quails (*Coturnix japonica*). Additionally, the release in the wild of Japanese quails or their hybrids with the European common quail is banned in several European countries. Indeed, the introgression of alleles from Japanese quails into the gene pool of wild populations of European common quails poses a serious threat to the genetic integrity and the long-term preservation of the native populations since the genetic dilution of alleles specific to the native quails may affect their behaviour and adaptability. Still, this study has shown that game-farmed quails display a more fearful behaviour than Japanese ones when confronted with human beings, and that high intra-species variability exists regarding the avoidance response of quails. In summary, these results support that a genetic basis exists for the fear response of quails and that, as long as genetic integrity of native populations is ensured, the selection of birds with fear and antipredator behaviour similar to that of quails born and living in the wild could be advantageous for game purposes.

## 1. Introduction

The European common quail (*Coturnix coturnix* Linnaeus, 1758) is a small migratory gallinaceous bird native to central and southern European countries. Its preferred breeding habitat encompasses grassland and cropland ecosystems [1,2]. Albeit still deemed a species abundant in Europe, the risk category of the European common quail was changed in 2021 from Least Concern to Near Threatened in the European Red List of Birds [3], mainly due to the decline of the breeding population in Spain. The fall in the number of free-living European common quails is attributed to the use of herbicides and insecticides in farmlands, which has led to decreased availability of weeds, seeds and insects, and to the fact that in southern Europe some areas are becoming unsuitable for quails because of getting drier and warmer, as well as due to the large-scale trapping of migrating quails in nets in Egypt [3,4,5].

Since European common quail is a game bird, quails have been raised in captivity for several decades in order to produce birds that can be then released in hunting preserves, in this way counterbalancing the reduction in the stock of European common quails in the wild and ensuring or increasing the number of hunting bags, often by means of put-and-take shooting [6,7,8,9,10]. Accordingly, the number of farms raising quails in Spain has increased from 318 to 2768 between 2007 and 2024 [11], a relevant proportion of which (more than 16%) produce quail for hunting purposes [10]. Nevertheless, it must be acknowledged that in most cases, the releases of captive-reared quails only meet the demand of hunters for quails but do not actually replenish the wild populations of *Coturnix coturnix* [12].

Genetic types of quail that are being farmed for the hunting market include the European common quail [6,13], the domestic Japanese quail (*Coturnix japonica* Temminck & Schlegel, 1849) and, more frequently, hybrids of both species, which are usually obtained by crossing female Japanese quails with European common quail males, and then successive generations are backcrossed with males of the European common species until what is called game-farmed quail is obtained [8,14,15,16,17]. Breeders of quails prefer raising these hybrids or even the Japanese quail (*Coturnix japonica*) rather than the European common quail because the former lay a greater number of eggs and are more docile and easier to handle [13,18,19,20]. Indeed, domestic Japanese quails have been selected for tameness over decades because birds that do not fear human beings enjoy greater welfare, do not injure themselves and yield better results [21,22,23]. However, a main drawback of both these domestic Japanese quails and the hybrid ones is that these birds display poor ability to fly, as compared with free-living European common quails, and are not really appreciated by hunters [24]. Furthermore, releasing Japanese quails or even hybrids between Japanese and European common quails in the wild is banned in several European countries, including Spain, because they are not native species [14,15,25,26]. As a matter of fact, this practice must be avoided since it entails a serious threat to the genetic integrity and the long-term preservation of the native populations of pure European common quails [7,27]. Introgression of alleles from domesticated Japanese quails into the gene pool of wild populations of European common quails is likely to impact on some behavioural traits of European common quails, such as the sexual calls or the migratory habit after the harvesting of cereal crops [28], and may be responsible for the loss of the natural instinct to incubate eggs in wild populations of quails since this has already occurred in domestic Japanese quails [29]. This would eventually result in an impairment of the adaptive potential of wild populations of European common quails and thereby in a reduction of their survival rate [28]. In actual fact, it has already been highlighted that the genotype of more than 80% of the quails reared in game farms in Spain includes alleles originating from domestic Japanese quails [8].

Moreover, the release of captive-reared quails has proven to fail in restocking the natural populations of European common quails due to the fact that the survival rate of the released quails not being hunted is very low. Farm-reared birds usually show low antipredator instinct and are unable to find food by themselves, or are less able to assimilate natural food since they have been raised in environments with an abundance of manufactured processed feed [30,31,32,33]. Furthermore, it has also been observed that frequent handling of quail chicks during their early life causes imprinting to caregivers, forming lasting attachments, which makes these quails show lower fear and avoidance responses when they are adults [34]. Reduced antipredator responses in game-farmed quails, in terms of freezing or escape behaviours, may also be due to learned helplessness [35] or lack of exposure to predators during the rearing phase [36], aspects that remain to be investigated.

Nonetheless, there is also scientific evidence supporting that the fear response of quails is genetically determined [37] and hence, selection for increased fearfulness or propensity to react when confronted with predators in quails could be possible [22,38]. Therefore, genetic variation in fear and avoidance responses could be expected between different species of quails and also among individuals within the same species of quail. The identification of quails that are more fearful could be useful in order to select as breeding adults in the game farms the genetically pure quails of the native species that would give rise to individuals more likely to survive on their own in the wild, and also to those more attractive as game birds. The duration of the tonic immobility reaction is usually used in poultry to assess the level of fearfulness [39]. Birds remaining immobile for a long time are considered animals with a high level of fearfulness [38]. The existence of a genetic variation in the duration of the tonic immobility reaction has also been demonstrated in quails [40]. Thus, variability among individuals for this behavioural trait exists in populations of quails. Moreover, the antipredator response of various game and domestic species has been assessed using approach tests with a simulated aerial predator [41,42,43] and ground-based approach tests with human beings [44,45,46]. These tests enable us to investigate the escape distance of birds in two situations that occur in the wild: when they face predators or hunters. Although studies with farm-reared game birds such as red-legged partridges (*Alectoris rufa*) and pheasants (*Phasianus colchicus*) have been conducted to investigate tonic immobility and predation simulation [47,48,49], no such research has been conducted to date on quails raised in game farms that are intended for release in hunting preserves. We hypothesise that game-farmed quails, having a genetic base from the European common quail, would show more fear and would flee earlier than domestic Japanese quails when approached by a human being or a simulated aerial predator.

Therefore, this study aimed at evaluating the antipredator response of both the game-farmed quail and the Japanese quail (*Coturnix japonica*) according to the following three parameters: the distance of escape from a human being, the distance of escape from a simulated aerial predator and the tonic immobility test.

## 2. Materials and Methods

### 2.1. Animals and Housing

This study was conducted at the experimental facilities of the Research and Teaching Farm of the Higher Technical School of Agricultural Engineering of the University of Seville (Spain). The study was approved by the Academic Planning Commission of the Higher Technical School of Agricultural Engineering of the University of Seville (approval code COA130117). Housing and husbandry of the experimental flock complied with the Spanish Royal Decree 53/2013 [50] and the Directive 2010/63/EU of the European Parliament and Council [51], both on the protection of animals used for scientific purposes.

Fifteen males (average body weight ± standard deviation at the beginning of the trial: 126.4 ± 6.28 g) and fifteen females (140.6 ± 11.94 g) of game-farmed quail and fifteen males (317.0 ± 24.88 g) and fifteen females (329.0 ± 30.38 g) of Japanese quail were used. The lower body weight of the game-farmed quails was due to their European common quail genetics. Quails from both batches were adults and were purchased at six weeks of age, one month before the start of the trial, from a game farm in Archidona (Málaga, Spain) in the case of the game quails and from a farm in Córdoba (Spain) that sold birds of various species for rural poultry farming in the case of the Japanese quails. The genetic background of the acquired game-farmed quails could not be confirmed with certainty because no genetic tests were performed to ascertain it. However, given the phenotypic closeness and the fact that in this farm, they had been breeding game quails for decades to restock hunting preserves of European common quails, it could be surmised that these quails had a high proportion of alleles from the European common quail. Japanese quails were of the domestic type with isabelle plumage colour. On their farms of origin, the quails were raised with limited human contact until they were four weeks old in closed brooder barns heated with infrared lamps. Subsequently, the Japanese quails were housed in cages, and the game-farmed quails were housed in open-air flight pens. In the experimental farm, quails were housed inside an enclosed barn with natural lighting and no environmental control (temperature or relative humidity) and kept individually in flat-deck cages measuring 40 cm × 34 cm × 18 cm (length × width × height). The birds were fed ad libitum a balanced commercial pelleted feed (21.5% crude protein, 6.7% crude fibre, 3.5% ether extract, 6.7% ash; Piensos Andaluces Equilibrados SL, Los Palacios, Spain) meeting the requirements for breeding quails [52]. Water was supplied ad libitum.

### 2.2. Trials and Experimental Design

Three tests were carried out with each animal (birds were subjected to each test once), at 10 weeks of age and on different days for each bird. Tests were performed in this order:

- Human approach test. This measured the distance at which the bird initiated the escape reaction when a human being approached in a straight line [53].
- Simulated aerial predator approach test. This test evaluated the antipredator behaviour of birds when a peregrine falcon model approached and flew in a straight line toward the bird at a height of 2 m [47].
- Tonic immobility test. This test assessed the birds’ fear reaction and consisted of measuring the time the bird remained motionless when placed on its back [54].

To carry out these tests, 15 quails of each sex (male and female) and genotype (game-farmed quail and Japanese quail) were used. These tests were conducted in the same barn where the quails were housed and were performed in the absence of people, noise and other animals not involved in the tests to avoid interference. A video camera (Panasonic Lumix DMC-FS35^®^; Panasonic Corporation, Kadoma Osaka, Japan) was used to record the birds’ reactions, and the ethogram coding was performed by the same person to avoid bias in the interpretation of behavioural criteria. A stopwatch (Fisherbrand Jumbo Traceable^®^; Fisher Scientific, Pittsburgh, PA, USA) was also used to measure the birds’ reaction times.

### 2.3. Human Approach Test

To conduct this test, the quail was placed in a 60 cm × 20 cm × 50 cm (length × width × height) wire mesh experimental cage that was open only at the front, the length of which was designed so that the birds would have enough space to walk on the floor, and which was narrow enough so that when the bird moved in response to the stimulus, it did so in a direction transverse to the observation direction, so that the observer could easily see this movement. The roof of the experimental cage, situated at a height sufficient to allow the bird to fly, was made of soft, loose plastic mesh to prevent startled quails from suffering neck and head injuries from banging their heads against the cage ceiling in their typical vertical flight response, which allows them to escape from predators in nature [55]. A 50-metre-long measuring tape was stretched across the floor of the barn, starting at the experimental cage. At the end of the tape, a polystyrene screen was placed that concealed the persons who performed the experiment. The screen had a small window measuring 25 cm high and 40 cm wide, allowing observation of the birds’ reactions.

To conduct the experiment with each quail, the bird was placed in the experimental cage, and the video camera and stopwatch were turned on synchronously. The testers then hid behind the screen for 10 min, which was considered sufficient time for the animal to calm down and relax after being placed in the experimental cage. After the 10 min wait, a person dressed in a white coat emerged from behind the screen and walked toward the experimental cage at approximately 1 m/s, observing and recording the bird’s reactions and the distances at which these occurred. The person walking toward the birds was always the same and had previously trained to standardise the walking speed.

### 2.4. Simulated Aerial Predator Approach Test

For this test, a silhouette of a peregrine falcon with outstretched wings (*Falco peregrinus* Tunstall, 1771), a raptor that usually preys on quail [56], was made from polystyrene and painted brown, measuring 60 cm in length and 90 cm in wingspan. The silhouette was fixed to a wooden crossbar, at the transverse ends of which eyebolts were placed to attach it to the guide cables, and at the longitudinal ends of which other eyebolts were placed to allow the passage of the cable that actuated its flight when the operator pulled on it. To fly the simulated aerial predator silhouette with the same trajectory for all the tested birds, a facility was built with the following characteristics: Three eyebolts were placed 2 m high on the wall behind the experimental cage, through which three cables were passed: two at the ends of the predator silhouette, separated by 70 cm, to guide its flight, and one in the centre to activate its flight using a pulley system. A 2 m high support was placed 15 m away from the experimental cage, from which the silhouette was flown toward the experimental cage. The cable used consisted of nylon rope that allowed the predator model to slide with low noise emission. A 15 m long measuring tape was stretched across the floor of the barn, starting at the experimental cage and ending at the support, which was opaque to conceal the person manually activating the flight of the predator silhouette. A screen was placed 3 m away from the experimental cage and in a position that allowed the flight of the simulated predator silhouette and the bird’s reactions to be monitored simultaneously. The quail and the silhouette were observed through the window as the latter began to fly, approaching the experimental cage. In this case, one person stood behind the screen acting as an observer and another one behind the support to make the silhouette fly by lifting the drive cable. The person operating the model’s flight was always the same and had previously trained to standardise the flight speed.

In the same way as in the human approach test, in this test, the bird was placed in the experimental cage, and the video camera and stopwatch were turned on synchronously. Before activating the simulated aerial predator silhouette’s flight, the bird was allowed to calm down for 10 min. After this time, the silhouette was flown toward the animal at approximately 1 m/s, and the observer, hidden behind the screen, recorded the animal’s reactions and the time at which these occurred.

### 2.5. Tonic Immobility Test

To place the quails subjected to this test, a wooden cradle was built that was formed by two slats of 25 cm length and 10 cm width, forming a 90° angle. The cradle was then secured to a table.

This test was carried out as follows. While one operator timed, recorded, and observed, the other performed the tonic immobility test by placing the bird on its back in the cradle. The following times and criteria were used to perform the tonic immobility test:

- The operator immobilised the quail for 10 s by placing a hand on the animal’s neck and the other hand on its chest with gentle pressure.
- After the 10 s restraint, the bird was released, and the time it remained motionless before standing up was recorded. The immobility test was considered valid if the bird remained motionless on its back for at least 15 s after release.
- The maximum number of test attempts per bird was five. If the bird did not remain motionless on the fifth attempt, the test was terminated, and the duration of immobility was considered zero seconds. The quail was considered to have stopped its immobility when it stood up, leaving its lying position on its back.
- The total time an animal was allowed to remain motionless was a maximum of 300 s after releasing it. If the animal did not stand up after this time, the test was interrupted, and the duration of the immobility was considered to be 300 s.
- When the bird stood up on its own before the maximum allowed time, the duration of immobility was recorded.

### 2.6. Variables Recorded

The variables measured in the human approach and simulated aerial predator approach tests were as follows:

- Distance (m) with respect to the human being or the simulated aerial predator at which the bird made a movement for the first time, by changing its location on the floor of the experimental cage (variable “Movement”).
- Distance (m) with respect to the human being or the simulated aerial predator at which the bird first performed the crouching reaction, by remaining motionless in its position with the body resting on the cage floor (variable “Crouching Down”).
- Distance (m) with respect to the human being or the simulated aerial predator at which the bird made its first attempt to take flight (variable “Flight”).
- Subsequently, the distance (m) at which the bird performed the first of the three reactions mentioned above was observed (variable “First Reaction”).

When a bird did not display any of those reactions, no record was assigned for that reaction to that bird.

The variables measured in the tonic immobility test were as follows:

- Number of attempts required to induce tonic immobility (variable “Number of Inductions”).
- Duration (s) of the tonic immobility response (variable “Duration of Immobility”).

### 2.7. Statistical Analyses

Levene’s test [57] was used to evaluate homogeneity of variance. Data of the parameters assessed in the three trials were subjected to an analysis of variance (ANOVA) with quail genotype and sex and their interaction as the main sources of variation by using the GLM procedure of SAS (Version 9.4, SAS Institute Inc., Cary, NC, USA). When interactions were found to be significant (*p* < 0.05), means of the experimental groups were compared using a *t*-test. Chi-square tests were used to compare the proportions of birds that flew in the human approach and in the simulated aerial predator tests, as well as to compare the proportions of birds whose first reaction was movement or crouching down according to their sex and genotype. The Pearson correlation coefficients between the variables measured in the three trials were obtained with the CORR procedure of SAS. The Hochberg method [58] was applied to make adjustments to *p*-values when testing pairwise comparisons in the correlation analysis. The quail represented the experimental unit for all the variables considered in this work.

## 3. Results

### 3.1. Human Approach Test

Table 1 shows the distance at which quails moved, crouched down, flew and performed the first out of the three previous reactions in the human approach test, according to quail genotype and sex.

The distance at which birds moved when a human being was approaching was greater for game-farmed quails than for Japanese quails (37.4 vs. 19.6 m, *p* < 0.001; Table 1). However, no significant difference between quail genotypes was found for the distance at which quails crouched (23.2 m, on average). When the human being got even closer, 10 out of the 30 game-farmed quails flew, whereas only 5 of the Japanese quails did so. However, the difference between the two genotypes in the percentage of quails that flew did not reach the significance level (*p* = 0.136). The average distance at which birds flew when a human being was approaching was 3.67 m, with no significant difference between quail genotypes. No differences due to sex were detected for the distances at which quails moved, crouched or flew. Finally, it can be pointed out that a great variability existed among individuals within each quail genotype for the distance at which quails moved. The coefficient of variation for the latter parameter amounted to 39.0 and 71.8% for the game-farmed and Japanese quails, respectively.

The distance at which birds performed the first of the previous reactions (movement, crouching down or flight) when a human being was approaching was greater for game-farmed quails than for Japanese quails (40.4 vs. 25.8 m, *p* < 0.001; Table 1). In this test, the first reaction of quails was movement or crouching, but never flight (Table 2). No effect of quail genotype or sex was found on the proportion of birds whose first reaction was movement or crouching down when the human being was approaching.

### 3.2. Simulated Aerial Predator Approach Test

Table 3 shows the distance (m) at which quails moved, crouched down, flew and performed the first out of the three previous reactions in the simulated aerial predator approach test, according to quail genotype and sex.

When the approach test was performed with a simulated aerial predator, no significant effect of either quail genotype or sex was found (Table 3) on the distance at which quails made a movement (8.94 m, on average). However, regarding the distance at which quails crouched, it was observed that female quails of the Japanese species were those crouching down before (at the longest distance with respect to the predator: 9.83 m), whereas no significant difference existed for this trait among the other three experimental groups (6.84 m, on average). The percentage of quails flying when the predator got closer was higher for the Japanese species than for the game-farmed quails (23.3 vs. 3.33%, *p* = 0.023). On the contrary, the percentage of quails flying did not differ significantly between sexes (*p* = 0.129). The average distance at which birds flew when a simulated aerial predator was approaching was 4.25 m, with no significant difference between quail genotypes or sexes.

Regarding the distance at which birds performed the first of the previous reactions (movement, crouching down or flight) when a simulated aerial predator was approaching, it was noticed that female quails of the Japanese species were those reacting before (at the longest distance with respect to the simulated aerial predator: 12.1 m), whereas no significant difference arose for this parameter among the other three experimental groups (9.67 m, on average). Again, also in this test, the first reaction of quails was movement or crouching down, but never flight (Table 4). No effect of quail genotype or sex was found on the proportion of birds whose first reaction was movement or crouching down when the simulated aerial predator was approaching. Nevertheless, a trend was found (*p* = 0.071) that indicated that in this test, the first reaction of males was mainly moving (63.3% of males), whereas crouching down was how most females reacted at first (60.0% of females).

### 3.3. Tonic Immobility Test

The results of the tonic immobility response of quails, according to their genotype and sex, are provided in Table 5.

Fewer inductions were needed to provoke the tonic immobility reaction in the game-farmed quails than in the Japanese ones (3.10 vs. 4.10, *p* = 0.009). However, the duration of the tonic immobility response did not differ significantly between the two genotypes of quails (82.0 s, on average). No significant influence of sex on the tonic immobility behaviour was noticed. As reported for the distance at which quails moved in the human approach test, also for the duration of the tonic immobility response, the intra-group coefficient of variation was higher for the Japanese quails than for the game-farmed quails (145 vs. 102%).

### 3.4. Correlations

Table 6 shows the correlations between the two main reactions of the quails in the human approach and simulated aerial predator tests and the parameters in the tonic mobility test.

A significant negative relationship was detected between the distance at which the quail moved when a human being approached and the number of inductions needed to cause the tonic immobility response (r = −0.267, *p* = 0.048). Furthermore, the number of inductions needed and the duration of the tonic immobility response were also negatively correlated (r = −0.551, *p* = 0.001).

## 4. Discussion

With particular impetus in the second half of the 20th century, the production of game birds in farms for release and restocking of hunting preserves experienced significant development in Europe [10,59,60,61] and in other regions of the world [62,63] to meet the increased demand for hunting and to recover declining wild populations of these game birds [64,65]. Although such captive production of game birds has successfully achieved its objectives in many cases [66,67,68], it has meant a change in the paradigm of game management, shifting in many territories from being based on the management of natural populations mainly through hunting regulation and habitat preservation and improvement [69,70] to a livestock-based and intensified model leading to releases and restocking with specimens raised in game farms [64,71]. Some of the problems that can arise from the captive breeding of game species intended for release and restocking are the potential introduction of diseases into the environment through the specimens released [72], the lower biological fitness of the captive-bred specimens when developing in the wild [28,73], the worse adaptation to natural food of farm-raised birds as a result of having been raised in artificial environments [30], lower survival rate due to predation [73], worse flying ability [74] and lower escape reaction to predators and hunters [75], as well as the risk of introgression of allochthonous genes when captive breeding is carried out through hybridisation and crossbreeding with non-native species and domestic breeds [8,73].

The production of quails in game farms intended for release in hunting preserves, mainly aimed at intensive shooting [10] rather than at recovering populations due to the migratory nature of quails that limits their settlement in the release territory [6], is carried out in Europe principally with quails crossed by means of an absorption cross between the European common quail and the domestic Japanese quail, and sometimes even directly with Japanese quails [10,17]. This practice, in addition to causing the introgression of allochthonous genes into native populations of European common quail [8,73], entails the release of specimens of uncertain hunting quality in terms of genetic integrity, flying ability and escape reaction from predators and hunters [76,77]. To the best of our knowledge, this is the first research work comparing antipredator behaviour between game-farmed quails and domestic Japanese quails.

Although there is some evidence about the effect of large animals having longer escape initiation distances than small animals because larger animals may be at greater risk due to lower escape speed or increased visibility [78], the human approach test carried out in this study showed that the game-farmed quails, although smaller, moved and performed their first reaction (movement or crouching down) at a greater distance from the human being than the Japanese quails. This implies a greater antipredator response of the former compared to the latter, which results from the fact that domestic quails have reduced their escape reaction due to tameness, a consequence of the domestication process [79]. This observation coincides with what was demonstrated in the game-farmed red-legged partridge by Binazzi et al. [47]. Thus, as it should have been expected, game-farmed quails showed more reactive behaviour than Japanese quails, especially when the tests performed implied interaction with human beings. Game-farmed quails moved at a further distance with respect to an approaching human being and needed less fear-inducing stimuli to display tonic immobility. Indeed, these two parameters were found to be significantly correlated, and the results obtained for both traits revealed that game-farmed quails were more frightened of human beings. Previous research works [37,80,81,82,83] have reported that more fearful birds become motionless earlier when subjected to the tonic immobility test. In the current study, it was also detected that a significant negative correlation existed between the number of inductions needed to prompt the tonic immobility reaction and the duration of the latter, as well as with the distance at which the quail moved when a human being approached. Our results are in line with those of Thompson et al. [84], who demonstrated that tonic immobility is a terminal defence mechanism elicited by predator contact that can reduce the probability of sustained attack by predators because the time spent by the predator stalking and attacking a quail is inversely related to the time the bird remains in tonic immobility. Likewise, in the studies published by Jones et al. [37] and Minvielle et al. [38], the correlation coefficient between the number of necessary inductions and the duration of the tonic immobility response of quails was –0.895 and –0.871, respectively. Nevertheless, in the present trial, the duration of the tonic immobility response did not differ significantly between the two genotypes of quails. Similarly, Mahmoud et al. [85] found significant differences among the treatments tested for the number of attempts needed in chickens to provoke tonic immobility, but then no significant effect of these same treatments was detected on the duration of the tonic immobility response. Both in the case of the present work and in the study of Mahmoud et al. [85], the fact that the differences observed among groups of birds for the tonic immobility duration did not reach the significance level could be attributed to the high intra-group variability existing for this parameter. For instance, the coefficient of variation for the duration of the tonic immobility reaction amounted to 124% in the present work. Nonetheless, the average values obtained in the current trial for the tonic immobility duration fall within the range of values reported in previous research conducted with unselected quails [39].

Regarding inter-individual variability, the coefficient of variation was found to be rather high in Japanese quails for the distance at which quails first moved when a human being was approaching and for the duration of the tonic immobility response (71.8% and 145%, respectively). High inter-individual variation in a human approach test conducted with farm-reared Japanese quails had previously been reported [46]. The high variability detected within the group of Japanese quails for the behavioural traits measured in the current study reflects an underlying genetic diversity [86] that, as observed, leads to a lack of consistency in the antipredator behaviour. This wide genetic diversity is of interest when aiming at selecting individuals differing positively for a particular trait [87] but may be unsuitable and undesired in birds raised to be released into the wild since birds used for replenishing wild populations should possess a gene pool similar to that of the native populations already inhabiting the wild ecosystem and should not introduce allochthonous alleles [27], as the genetic integrity of the native flocks should be safeguarded. Hence, this justifies the ban on releasing Japanese or hybrid quails into the wild in European countries.

The lack of significant effect of quail sex on variables such as those measured in the human approach test or those of the tonic immobility test is consistent with previous findings. Accordingly, the absence of differences due to sex in the tonic immobility test was reported by Mills and Faure [88] and Minvielle et al. [38]. Furthermore, as reviewed by Mills et al. [34], in most cases, the fear and avoidance responses of quails do not differ between males and females.

The fact that limited differences were found in this work between game farm-raised quails and Japanese quails for the antipredator response to a simulated aerial predator could be ascribable to the fact that fear and escape reactions to birds of prey are strongly conserved even in poultry breeds despite their long history of domestication, as it has been widely reported in the literature [89,90].

The interaction observed in the simulated aerial predator approach test between genotype and sex of quail, consisting in female Japanese quails crouching down and performing a first reaction (crouching down or movement) at a greater distance from the stimulus than males, while there was no difference between sexes in game-farmed quails, could be due to the fact that the domestication experienced by the Japanese quail has led to greater variability between sexes in the response to birds of prey. This finding is in line with what was reported by Axling [91], who observed in chickens an interaction between genotype effect and sex effect for variable stand alert in the aerial predator test, in a trial comparing wildtype birds and mutants for the thyrotropin receptor gene (responsible for differences in individual stress response). In fact, in quail, sex differences in the thyrotropin receptor stress response are influenced by steroid hormone receptors and their interactions with the stress axis, particularly the hypothalamic–pituitary–adrenal axis [92]. Our results also suggest a loss, in Japanese quails, of the alert reaction characteristic of male birds [93] due to domestication itself [29] and the lack of experience and stimuli facing aerial predators during the rearing period [36]. In fact, to improve hunting quality during game bird breeding, alternative rearing systems are being proposed and developed in which birds are raised with parental tutors [94,95] and are challenged with predator models [96], with the aim of strengthening the birds’ innate response through learning and experience.

The fact that in both the human approach test and the simulated aerial predator approach test, the quails’ first reaction was crouching down or movement but never taking flight was consistent with the typical behaviour of steppe galliformes, which, although they fly well, have predominantly terrestrial movement habits [97] and primarily freezing responses to the presence of predators [47], especially if the latter are birds of prey [96], and they only fly short distances [98]. They also benefit from their ability to hide from predators due to their camouflage plumage, which is particularly effective in the case of quail in both sexes [97] because these game birds live in open spaces with vegetation and bushes that can be used by birds as camouflage to avoid being detected by predators [47].

The differences observed in the present study between the responses of quails in the human approach test and the simulated aerial predator approach test could have been due to the well-known circumstance that galliformes discriminate between aerial and ground predators, producing distinctive antipredator responses [99]. This was particularly evident for the distance at which the quails moved, crouched down or performed the first of these two reactions, which was three times greater in the human approach test than in the simulated aerial predator approach test. Our results, however, do not coincide with those of Buchwalder and Wechsler [100], who reported that presenting Japanese quail with a frightening stimulus above the birds elicited flight behaviour more frequently than when the same stimulus was presented at the side of the birds. This discrepancy may stem from the fact that in our study, the stimulus presented was different in the human approach test and in the simulated aerial predator approach test.

Game-farmed quails display greater antipredator response than domestic Japanese quails, even though they are also raised in farms from hatching. Therefore, the techniques used to raise birds in game farms, which consist in rearing them on the ground from birth, first in brooder barns and then in open-air flight pens with limited human contact [6,10,17], are successful, at least partially, in making birds reproduce some attributes of the wild character of free-living quails.

A potential limitation of this study lies in the fact that the two experimental groups of quail came from two different farms with some differences in early-life environmental conditions and bird handling, which derive from the fact that game-farmed quails are raised so as to prepare them for release, while Japanese quail are raised for meat or eggs. Indeed, the goal was to investigate the differences in the antipredator response of both types of quail, derived not only from their different genetics but also from their rearing systems. In any case, if the aim is to investigate potential differences in antipredator response due solely to the genotype of the birds, both types of quail would have to be raised on the same farm, subjected to the same handling and submitted directly to the test performed, which could be the subject of further research. Although the human approach and simulated aerial predator tests were standardised and followed methodologies usual in this research field [47,53], another potential limitation of this study could rely on the limited ecological realism of the experimental setting, which might not fully replicate ecologically valid predation or hunting scenarios. Therefore, further research could attempt to replicate conditions more similar to quail predation in natural conditions or in which quails face hunters in the field. Furthermore, to further investigate the potential genetic influence suggested by the results obtained, it would be useful to perform genetic analyses and heritability estimates. The latter aspect becomes particularly relevant in game-farmed quails because, as they are usually obtained from successive generations of backcrossing hybrid quails (*Coturnix coturnix* × *Coturnix japonica*) with males of the European common quail species, the proportion of European common quail alleles in the game-farmed quails can vary across farms. Moreover, in the tonic immobility test, observer bias could have occurred because of the observer not being blinded when handling the quails. Indeed, the phenotype and weight of the Japanese and game-farmed quails are very different, making them easily identifiable, and this could have influenced the interpretation of the results. Lastly, since all birds were subjected to the three behavioural tests in the same fixed order, potential carry-over or habituation effects are not to be fully excluded. Although the tests were conducted on separate days, prior exposure to one type of stimulus (e.g., human approach) may have induced reactivity in subsequent tests (e.g., simulated aerial predator or tonic immobility). Nevertheless, it should also be pointed out that these alleged habituation effects would have occurred in all the experimental groups since they were treated the same. Therefore, future studies should consider randomising test order or controlling for sequential effects.

In light of the results of this study and with a view to achieving the production in game farms of more biologically efficient quails after release, further lines of research could investigate and compare the antipredator response among free-living European common quails, purebred European common quails raised in captivity and the crossbred quails currently being raised in game farms. Furthermore, it would also be of interest to investigate the flying ability of quails raised in game farms. These aspects have been the subject of research in other game species bred in captivity, such as red-legged partridges and pheasants [48,101,102], but not in quails. Also in view of the relatively small difference in antipredator response between game-farmed quail and Japanese quail shown in the present study, probably due to the introgression of domestic genes into the gene pool of game-farmed quails [8,9,10,11,12,13,14,15,16,17,18,19,20,21,22,23,24,25,26,27,28], it would be advisable to enhance the development of a breeding technology for genetically pure native European common quails in game farms, a knowledge area in which little research has been conducted to date [13,18,26,103,104,105,106,107].

## 5. Conclusions

When subjected to tests that implied interaction with human beings, game-farmed quails displayed greater escape initiation distances and required fewer inductions to exhibit tonic immobility than Japanese ones. Additionally, high intra-species variability existed for the distance at which quails moved when a human being was approaching and for the duration of the tonic immobility response. Consequently, these results suggest the presence of a genetic component in the fear and antipredator response of quails and, therefore, more reactive birds could be reared for game purposes. No sex differences were observed in the human approach and tonic immobility tests. These two tests were significantly correlated since a significant negative relationship was found between the distance at which quails moved in the human approach test and the number of attempts needed to provoke the tonic immobility response, which suggests convergent validity between the two fear-assessment paradigms.

Female Japanese quails crouched down earlier in response to aerial predator stimuli, whereas the distance at which quails crouched down in this test did not differ among the other experimental groups of birds.

In the approach tests performed, the first reaction of quails was either moving or crouching down, but never taking flight.

## Figures and Tables

**Table 1:** Distance (m) at which quails moved, crouched down, flew and performed the first out of the three previous reactions in the human approach test, according to quail genotype and sex. Sample size: 15 birds per quail genotype and sex. N (%): number and percentage of birds displaying each specific reaction.

	Movement (m)		Crouching Down (m)		Flight (m)		First Reaction (m)	
	N	Mean	N	Mean	N	Mean	N	Mean
Quail genotype								
Japanese	27 (90.0)	19.6	25 (83.3)	21.5	5 (16.7)	6.20	30 (100)	25.8
Game-farmed	29 (96.7)	37.4	28 (93.3)	24.8	10 (33.3)	2.40	30 (100)	40.4
Sex								
Male	28 (93.3)	26.4	26 (86.7)	23.2	5 (16.7)	2.80	30 (100)	31.7
Female	28 (93.3)	31.2	27 (90.0)	23.2	10 (33.3)	4.10	30 (100)	34.5
Quail genotype × Sex								
Japanese × Male	13 (86.7)	16.1	11 (73.3)	19.6	0 (0.0)	-	15 (100)	23.1
Japanese × Female	14 (93.3)	22.9	14 (93.3)	23.0	5 (33.3)	6.20	15 (100)	28.4
Game-farmed × Male	15 (100)	35.4	15 (100)	25.9	5 (33.3)	2.80	15 (100)	40.3
Game-farmed × Female	14 (93.3)	39.5	13 (86.7)	23.5	5 (33.3)	2.00	15 (100)	40.5
Overall mean		28.8		23.2		3.67		33.1
SEM		3.77		4.23		2.84		3.57
*p*-value								
Quail genotype		<0.001		0.472		0.296		<0.001
Sex		0.173		0.940		0.845		0.441
Quail genotype × Sex		0.733		0.521		-		0.486

SEM: Standard error of the mean.

**Table 2:** Contingency table of the proportion of birds whose first reaction was movement or crouching down in the human approach test, according to quail genotype and sex (N = 30).

	Movement		Crouching Down		*p*-Value
	N	%	N	%	
Quail genotype					
Japanese	20	66.7	10	33.3	0.390
Game-farmed	23	76.7	7	23.3	
Sex					
Male	20	66.7	10	33.3	0.390
Female	23	76.7	7	23.3	

**Table 3:** Distance (m) at which quails moved, crouched down, flew and performed the first out of the three previous reactions in the simulated aerial predator approach test, according to quail genotype and sex. Sample size: 15 birds per quail genotype and sex. N (%): number and percentage of birds displaying each specific reaction.

	Movement (m)		Crouching Down (m)		Flight (m)		First Reaction (m)	
	N (%)	Mean	N (%)	Mean	N (%)	Mean	N (%)	Mean
Quail genotype								
Japanese	22 (73.3)	9.77	23 (76.7)	7.96	7 (23.3)	4.14	30 (100)	10.4
Game-farmed	26 (86.7)	8.23	26 (86.7)	7.31	1 (3.33)	5.00	30 (100)	10.1
Sex								
Male	25 (83.3)	8.72	24 (80.0)	6.79	6 (20.0)	4.17	30 (100)	9.70
Female	23 (76.7)	9.17	25 (83.3)	8.40	2 (6.67)	4.50	30 (100)	10.9
Quail genotype × Sex								
Japanese × Male	11 (73.3)	8.82	11 (73.3)	5.91 ^b^	5 (16.7)	4.00	15 (100)	8.73 ^b^
Japanese × Female	11 (73.3)	10.7	12 (80.0)	9.83 ^a^	2 (6.67)	4.50	15 (100)	12.1 ^a^
Game-farmed × Male	14 (93.3)	8.64	13 (86.7)	7.54 ^ab^	1 (3.33)	5.00	15 (100)	10.7 ^ab^
Game-farmed × Female	12 (80.0)	7.75	13 (86.7)	7.08 ^ab^	0 (0.0)	-	15 (100)	9.60 ^ab^
Overall mean		8.94		7.61		4.25		10.3
SEM		1.24		0.937		0.408		1.08
*p*-value								
Quail genotype		0.274		0.535		0.308		0.782
Sex		0.777		0.131		0.437		0.283
Quail genotype × Sex		0.321		0.040		-		0.043

^a,b^ Means in the same column accompanied by different superscript letters are significantly different (*p* < 0.05). SEM: Standard error of the mean.

**Table 4:** Contingency table of the proportion of birds whose first reaction was movement or crouching down in the simulated aerial predator test, according to quail genotype and sex (N = 30).

	Movement		Crouching Down		*p*-Value
	N	%	N	%	
Quail genotype					
Japanese	15	50.0	15	50.0	0.796
Game-farmed	16	53.3	14	46.7	
Sex					
Male	19	63.3	11	36.7	0.071
Female	12	40.0	18	60.0	

**Table 5:** Number of inductions to provoke the immobility response and duration (s) of the immobility response in the tonic immobility test in quails according to their genotype and sex. Sample size: 15 birds per quail genotype and sex.

	N	Number of Inductions (n)	Duration of Immobility (s)
		Mean	Mean
Quail genotype			
Japanese	30	4.10	79.4
Game-farmed	30	3.10	84.5
Sex			
Male	30	3.87	85.5
Female	30	3.33	78.4
Quail genotype × Sex			
Japanese × Male	15	4.40	78.1
Japanese × Female	15	3.80	80.7
Game-farmed × Male	15	3.33	92.9
Game-farmed × Female	15	2.87	76.1
Overall mean		3.60	82.0
SEM		0.370	27.1
*p*-value			
Quail genotype		0.009	0.850
Sex		0.155	0.794
Quail genotype × Sex		0.858	0.722

SEM: Standard error of the mean.

**Table 6:** Pearson correlation coefficients ^1^ between the variables measured in the present study.

		Human Approach Test		Simulated Aerial Predator Approach Test		Tonic Immobility Response	
		The Distance at Which the Quail Moved	The Distance at Which the Quail Crouched	The Distance at Which the Quail Moved	The Distance at Which the Quail Crouched	Number of Inductions Needed	Duration of the Tonic Immobility Response
**Human Approach Test**	The distance at which the quail moved		r = 0.068 *p* = 0.96	r = 0.007 *p* = 0.96	r = 0.075 *p* = 0.96	r = −0.267 *p* = 0.048	r = 0.042 *p* = 0.96
	The distance at which the quail crouched	r = 0.068 *p* = 0.96		r = −0.095 *p* = 0.96	r = 0.103 *p* = 0.96	r = −0.072 *p* = 0.96	r = 0.027 *p* = 0.96
**Simulated Aerial Predator Approach Test**	The distance at which the quail moved	r = 0.007 *p* = 0.96	r = −0.095 *p* = 0.96		r = 0.054 *p* = 0.96	r = −0.098 *p* = 0.51	r = 0.028 *p* = 0.96
	The distance at which the quail crouched	r = 0.075 *p* = 0.96	r = 0.103 *p* = 0.96	r = 0.054 *p* = 0.96		r = −0.149 *p* = 0.96	r = 0.183 *p* = 0.96
**Tonic Immobility Response**	Number of inductions needed	r = −0.267 *p* = 0.048	r = −0.072 *p* = 0.96	r = −0.098 *p* = 0.96	r = −0.149 *p* = 0.96		r = −0.551 *p* = 0.001
	Duration of the tonic immobility response	r = 0.042 *p* = 0.96	r = 0.027 *p* = 0.96	r = 0.028 *p* = 0.96	r = 0.183 *p* = 0.96	r = −0.551 *p* = 0.001	

^1^ N = 60 quails.

## Data Availability

The data supporting the conclusions of this article will be made available by the authors upon reasonable request.
